# AgingPLUS: Examining the Malleability of Implicit Views of Aging

**DOI:** 10.1093/geroni/igab046.1684

**Published:** 2021-12-17

**Authors:** Heidi Tseng, Garrett Forsyth, Abigail Nehrkorn-Bailey, Diana Rodriguez, Kat Thompson, Manfred Diehl

**Affiliations:** Colorado State University, Fort Collins, Colorado, United States

## Abstract

AgingPLUS also examines whether the intervention can change participants’ implicit VOA. To that end, participants completed a lexical decision-making task (LDMT) and the Brief Implicit Association Test (BIAT) at baseline and post-intervention. One-way ANCOVAs with baseline scores as covariate were used for these analyses. For LDMT, there was no significant difference between the groups regarding their post-intervention latencies for old-positive words, F(1,181) = 0.01, p = .60, old-negative words, F(1,181) = 0.43, p = .51, young-positive words, F(1,181) = 0.19, p = .67, and young-negative words, F(1,181) = 1.16, p = .28. For BIAT, both groups showed a slight preference for the young at baseline (mean d = 0.39), and post-intervention (mean d = 0.38). There was no significant difference between the groups regarding post-intervention d scores, F(1,181) = 0.002, p = .97. These preliminary findings suggest that in the current subsample, AgingPLUS did not significantly change participants’ implicit VOA.

